# Global Burden and Challenges of Melioidosis

**DOI:** 10.3390/tropicalmed3010013

**Published:** 2018-01-29

**Authors:** David AB Dance, Direk Limmathurotsakul

**Affiliations:** 1 Lao-Oxford-Mahosot Hospital-Wellcome Trust Research Unit (LOMWRU), Vientiane, Laos; 2 Centre for Tropical Medicine & Global Health, University of Oxford, Oxford OX3 7FZ, UK; 3 Faculty of Tropical Medicine and Infectious Diseases, London School of Hygiene and Tropical Medicine, London WC1E 7HT, UK; 4 Mahidol-Oxford Research Unit (MORU), Bangkok 10400, Thailand; 5 Faculty of Tropical Medicine, Mahidol University, Bangkok 10400, Thailand

Melioidosis, an infectious disease caused by the environmental bacterium *Burkholderia pseudomallei*, has remained in the shadows for far too long. Described over 100 years ago by Alfred Whitmore in Rangoon [1], the disease is so neglected that it is not even on any of the lists of neglected tropical diseases, despite the fact that it probably kills more people each year than diseases that are much better known, such as leptospirosis and dengue [2]. We aim to set the record straight.

In the first few years after its discovery, melioidosis was considered a relatively rare infection confined to areas where colonial medical services had been established, for example by the British in Burma (now Myanmar) [1], Ceylon (now Sri Lanka) [3] and the Federated Malay States (now Malaysia) [4], the French in Indochina (now Cambodia, Laos and Vietnam) [5,6,7], and the Dutch in the Dutch East Indies (now Indonesia) [8,9,10]. It was the French in Indochina who proved that the organism was a saprophyte rather than a zoonosis as had originally been suspected [11,12]. It was discovered for the first time in northern Australia in 1949 [13], although it appears that this is really where it actually originated [14]. *B. pseudomallei* appears to have spread from there to southeast Asia, and thence to Africa and the Americas [15,16]. The disease gained brief notoriety as a cause of infection amongst French and American troops serving in Southeast Asia [17,18,19]. Its unusual ability to remain latent after acquisition and cause a fatal disease many years later has given rise to the nickname ‘Vietnam Time Bomb’ [20]. *B. pseudomallei* has more recently been categorised as a ‘Tier 1 Select Agent’ because of its biothreat potential (https://www.ecfr.gov/cgi-bin/retrieveECFR?gp=&SID=8a4be60456973b5ec6bef5dfeaffd49a&r=PART&n=42y1.0.1.6.61).

Undoubtedly, it was the work of the Infectious Disease Association of Thailand that led to the recognition that melioidosis was actually a greatly-underestimated public health problem in some parts of the world. In 1985, they organised a meeting devoted to melioidosis that highlighted 686 cases of the disease occurring in Thailand over a relatively short time period [21]. This heralded a new wave of interest in the disease that has culminated in this current special issue of Tropical Medicine and Infectious Diseases. Writing in 1991, after spending 4 years observing what an important disease melioidosis was in northeast Thailand [22], one of us reviewed existing evidence and suggested that the disease was probably far more common worldwide than was currently appreciated [23]. This was not a new idea, as Fournier had made similar suggestions some 3 decades earlier [24,25]. The reasons for its under-recognition are a lack of diagnostic microbiology laboratories serving the rural poor in the tropics, who are most likely to acquire melioidosis, and a lack of familiarity and awareness amongst medical and laboratory staff, where such laboratories are available. More recently, the prediction has been vindicated by growing numbers of reports of the disease in new places, and increasing recognition within known endemic areas [26,27]. In 2016, the first attempt was made to estimate the global burden of human melioidosis in terms of cases and deaths, the resulting prediction being 165,000 and 89,000 per year, respectively, a mortality burden similar to that of measles [2].

Now, two years on from this modelling study [2], the time is right to take stock of what we have learned since then. In this issue, we have combined articles from countries and regions around the world that summarise the current status, including what is known locally about the burden of melioidosis, and the key challenges facing local clinicians, laboratory staff and public health and policy makers, in relation to this elusive but common and fatal disease. We hope that this will become a key source of information for those who share our concern and are taking actions against this disease.
